# Enhanced wound healing potential of arabincoside B isolated from *Caralluma Arabica* in rat model; a possible dressing in veterinary practice

**DOI:** 10.1186/s12917-024-04128-2

**Published:** 2024-06-29

**Authors:** Mawada Mohamed Ali, Asmaa Khairy Al-Mokaddem, Essam Abdel-Sattar, Riham A. El-Shiekh, Michael M. Farag, Samira H. Aljuaydi, Iman B. Shaheed

**Affiliations:** 1 Department of Pathology, Faculty of Veterinary Medicine, Cairo University, Giza, 12211 Egypt; 2 Department of Pharmacognosy, Faculty of Pharmacy, Cairo University, Cairo, 11562 Egypt; 3 Department of Pharmaceutics & Industrial Pharmacy, Faculty of Pharmacy, Cairo University, Cairo, 11562 Egypt; 4 Department of Biochemistry and Molecular Biology, Faculty of Veterinary Medicine, Cairo University, Giza, 12211 Egypt

**Keywords:** Wound healing, Histopathology, *Caralluma Arabica*, Arabinoside B

## Abstract

**Background:**

Wound management is a critical procedure in veterinary practice. A wound is an injury that requires the body’s cells’ alignment to break down due to external assault, such as trauma, burns, accidents, and diseases. Re-epithelization, extracellular matrix deposition, especially collagen, inflammatory cell infiltration, and development of new blood capillaries are the four features that are used to evaluate the healing process. Using a natural extract for wound management is preferred to avoid the side effects of synthetic drugs. The current study aimed to assess the effect of major pregnane glycoside arabincoside B (AR-B) isolated from *Caralluma arabica* (C. arabica) for the wound healing process.

**Method:**

AR-B was loaded on a gel for wound application. Rats were randomly distributed into six groups: normal, positive control (PC), MEBO®, AR-B 0.5%, AR-B 1%, and AR-B 1.5%, to be 6 animals in each group. Wounds were initiated under anesthesia with a 1 cm diameter tissue needle, and treatments were applied daily for 14 days. The collected samples were tested for SOD, NO, and MDA. Gene expression of VEGF and Caspase-3. Histopathological evaluation was performed at two-time intervals (7 and 14 days), and immunohistochemistry was done to evaluate α -SMA, TGF-β, and TNF-α.

**Result:**

It was found that AR-B treatment enhanced the wound healing process. AR-B treated groups showed reduced MDA and NO in tissue, and SOD activity was increased. Re-epithelization and extracellular matrix deposition were significantly improved, which was confirmed by the increase in TGF-β and α -SMA as well as increased collagen deposition. TNF-α was reduced, which indicated the subsiding of inflammation. VEGF and Caspase-3 expression were reduced.

**Conclusion:**

Our findings confirmed the efficiency of AR-B in enhancing the process of wound healing and its potential use as a topical wound dressing in veterinary practice.

**Supplementary Information:**

The online version contains supplementary material available at 10.1186/s12917-024-04128-2.

## Background

A wound is a disruption of the surface’s epithelium. It could be either superficial or complex. It is considered a deep wound involving vessels, tendons, nerves, or other structures [1]. Improvement of damaged epithelial tissue of the skin is known as wound healing. The wound healing process goes through 4 stages, including hemostasis, inflammation, proliferation, and remodeling [2].

The master key that organizes wound healing is the immune system. Once the skin gets injured, the 1st stage of wound healing (hemostasis) begins, and platelets aggregates under the effect of Transforming Growth Factor (TGF-β1, TGF-β2, and Platelets Derived Growth Factor (PDGF) chemotactic factors. Then, 2nd stage (inflammation), neutrophils, lymphocytes, and macrophages infiltrate the wound’s area and release various cytokines and chemokines to prevent the wound’s infection. At the same time, the final two stages (proliferation and remodeling) are done by growth factors released by keratinocytes, macrophages, platelets, and endothelial cells [3].

Owing to the frequent occurrence of wounds in veterinary patients, proper wound management is critical, and perfect healing is an important goal. It is vital to understand the mechanism of wound healing and the complex interaction between the cytokines and different cells [4]. In animals, improper wound management could result in serious problems; for example, in horses, ‘proud flesh’ is formed due to exaggerated granulation tissue formation, resulting in delayed healing [5]. In cats, a type of pseudo-healing describes superficial incision healing with a lack of healing in the underlying tissue [6]. One of the most important research areas is enhancing the normal wound healing process. That important goal has been served by various new dressings or other medicinal methods [7]. Natural wound dressing materials are preferred to avoid the adverse side effects of other synthetic chemicals, such as NSAIDs, resulting in delayed bone healing in dogs [8]. In veterinary practice, there are numerous new interventions used for improving wound healing. The most used included interactive dressings, which include films, foams, hydrogels, hydrocolloids, and alginates. Apart from these, there are other new inventions such as bioactive dressings like cellular mediators and growth factors, as well as some tissue engineering dressings like biosynthetic fish skin grafts [9].

Plant extracts and natural products are the primary healthcare sources for most of the world’s medicines. More than 400 plants have been identified as potentially useful alternative medicines for wound healing [10, 11].

Some plants of medical importance contain antibacterial and antifungal compounds that promote better wound healing. Some plants aid in the different stages of wound healing, some others help in the inflammatory phase by eliminating ROS and increasing antioxidant effects, in addition to decreasing chemical mediators such as tumor necrosis factor-α (TNF-α), interleukin-6 (IL-6), and inducible nitric oxide synthase (iNOS). Other medicinal plants aid in the angiogenesis, while other substances stimulate re-epithelialization and remodeling by increasing the expression of TGF-β. Some also aid in accelerating the wound healing process by promoting granulation tissue formation and collagen deposition during the proliferation phase [12].

Pregnanes and pregnane glycosides are well-documented as potential secondary metabolites with diverse biological activities, including immunomodulator, antarthritic, antiulcer, anti-nociceptive, anti-inflammatory, and antibacterial properties, distributed mainly in the families Apocyanaceae and Asclepiadaceae. Genus *Caralluma* R.Br. is commonly known for the presence of pregnane glycosides [13].

**Fig. 1:**
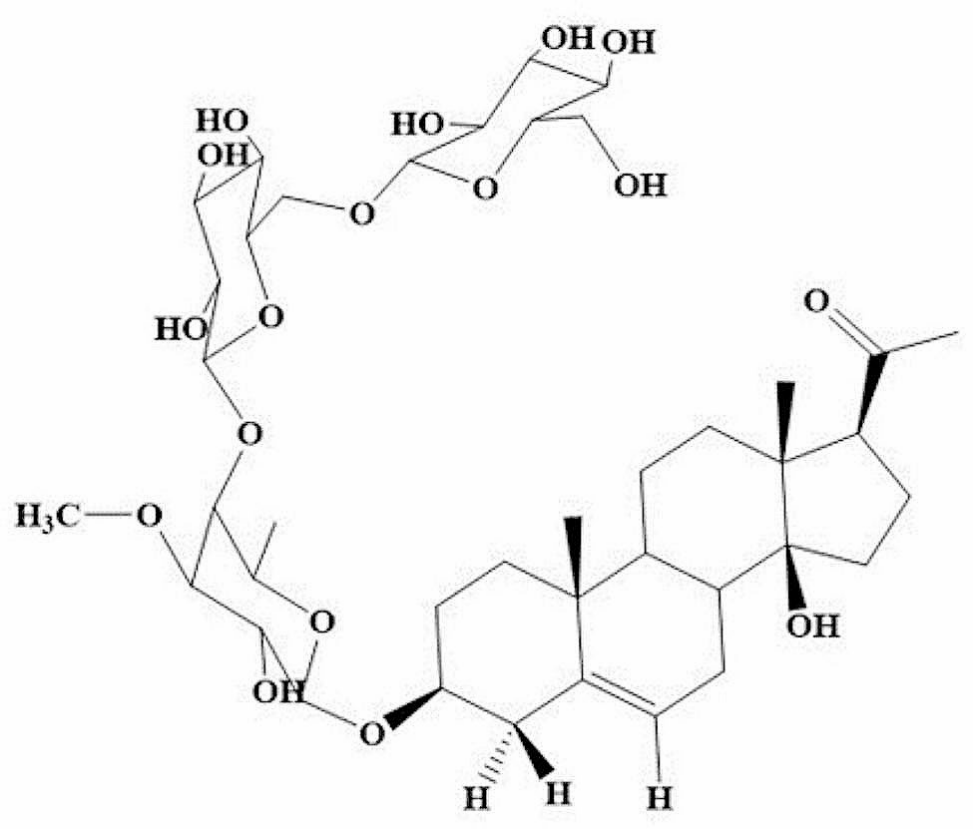
Chemical structure of arabincoside B

*Caralluma arabica* (*C. arabica*) is an herb widely distributed throughout Yemen, the United Arab Emirates, Oman, Saudi Arabia, and the Horn of Africa. In the Arabian Peninsula, a decoction of *C. arabica* is traditionally used to treat several skin conditions, including cuts, wounds, burns, and itchy skin [14]. In this context, isolation of the major pregnane glycoside arabincoside B (AR-B, C_40_H_64_O_17_) [15] (Fig. 1) becomes a mandatory concern to investigate its potential for wound healing properties and get insight into its mechanism of action for the first time.

It was mentioned by [16] that topical formulation is a practical and useful method that facilitates the healing process. In this context, we used Sodium carboxymethyl cellulose (NaCMC), a natural polymer known for its strong affinity for water.

This study aimed to study the effect of AR-B isolated from *C. arabica* as a natural material for enhancing the process of wound healing in rat models. So that it could be used as natural wound dressing material for wound management in veterinary practice.

## Results

### Characterization of arabincoside B

**Fig. 2:**
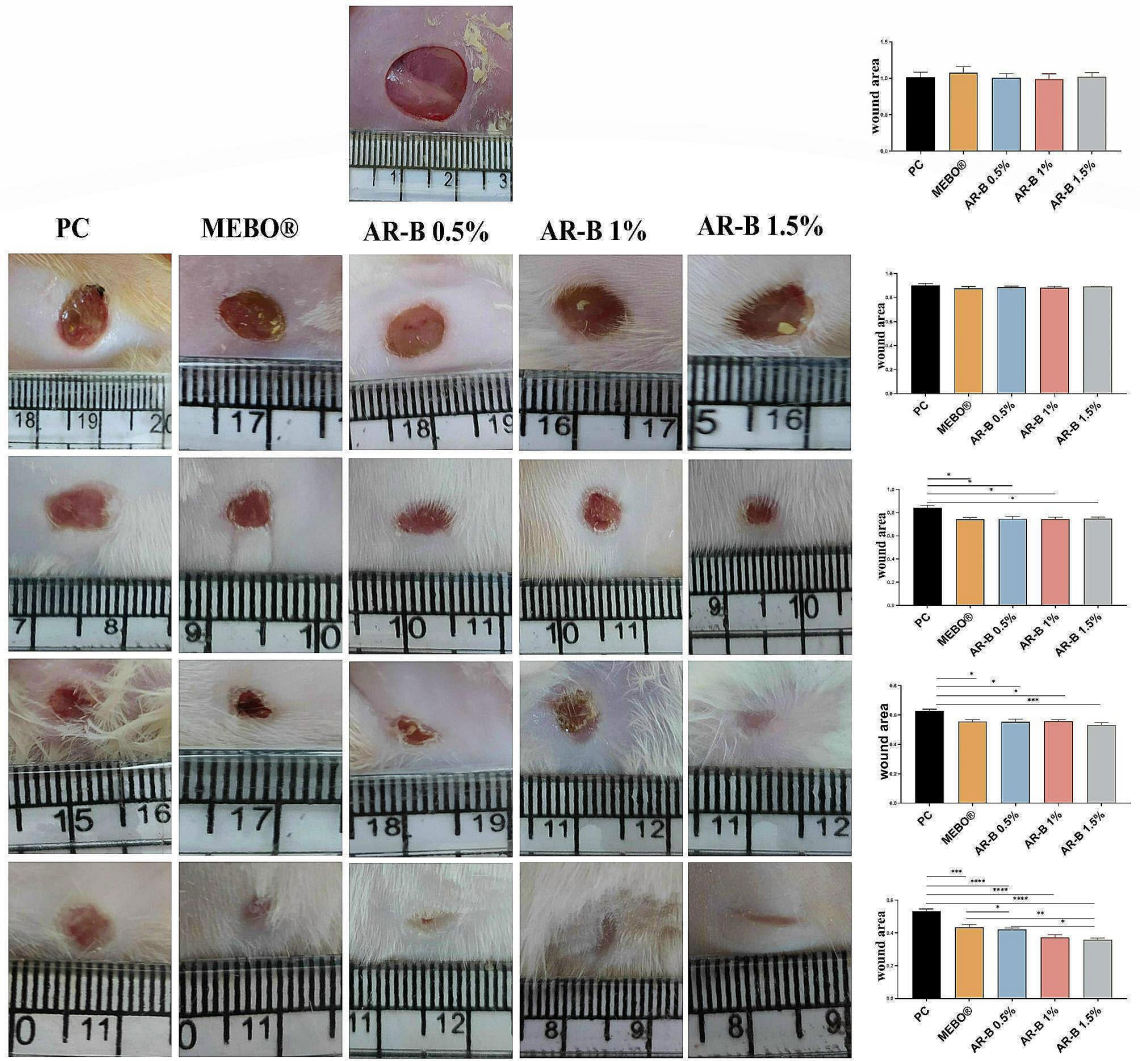
Photos of skin showing the wound area at the different time points. The first row represents 0-day, the 2^nd^ row represents 3 days post induction, the 3^rd^ row represents 7 days post induction, 4^th^ row represents 10 days post induction and 5^th^ row represents 14 days post induction. Charts represent wound area. Data are presented as mean ± SE. significant difference is considered at *P* < 0.05. (*) = summary of significance level and (ns) = non-significant

The structure of AR-B was elucidated using spectral means (IR, 1D & 2D-NMR, & ESI-MS). [α]D21 -98.52 (c. 0.10, MeOH); IR υmax (KBr, cm^-1^): 3379, 2935, 1681, 1419, 1361, 1346, 1284, 1176, 1049, 964, 763 and 640; Table 1.S. for ^1^ H and ^13 ^C NMR arabincoside B (Buker High-Performance Avance III FT-NMR spectrometer, 400 MHz, 100 MHz, CH3OH-d6, respectively) of aglycone and sugar moieties; Fig. (1.S) and Fig. (2.S) for ^1 ^H and ^13 ^C NMR spectra of arabincoside B; ESI–MS, *m/z* (rel. int.): 839.1 [M + Na]^+^ (100) in positive mode, and 815.0 [M-H]-in negative mode.

### Wound area

On the first day, all groups had no noticeable difference in the wound area. By the third day, the diameter had decreased, but no significant difference existed between the different treated groups. On the seventh day, the wound area was significantly decreased in all treated groups compared to the PC group. The same results were observed on the tenth day, with a more significant decrease in the wound diameter for the AR-B 1.5% group compared to the PC. By the 14^th^ day, all treated groups had shown significant improvement in a dose-dependent manner. There was also a significant difference between the MEBO® treated group and the AR-B 1% and AR-B 1.5% groups. However, there was no significant difference between the AR-B 1% and AR-B 1.5% groups (Fig 2).

### Oxidant and antioxidants evaluation

**Fig. 3:**
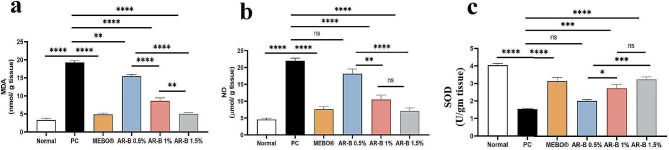
Charts represented (**a**) Malonaldehyde (MDA), (**b**) Nitic oxide (NO) and (**c**) Super oxide dismutase (SOD). Significant difference at *P* < 0.05. (*) = summary of significance level and (ns) = non-significant

Estimated MDA, NO, and SOD in wound tissue were illustrated in Fig. 3. MDA level was significantly decreased in the AR-B 1.5% treated group (5.03) compared to other AR-B treated groups (15.5 and 8.66 respectively). PC group (19.29) exhibited the highest level of MDA. There was no significant difference between the AR-B 1.5% group (5.03) and the normal and MEBO® treated (4.8) groups.

The level of NO was significantly decreased in the group treated with AR-B 1.5% (7.12) compared to those treated with AR-B 0.5% (18.19) and PC (21.99). However, no statistically significant difference existed between the group treated with AR-B 1% (10.49) and the AR-B 1.5% (7.12) group. Furthermore, there was no statistical difference between the normal (4.57) and MEBO® (7.61) groups compared to the AR-B 1.5% group.

SOD level was decreased significantly in the PC group (1.54); meanwhile, both the AR-B 1% (2.73) and AR-B 1.5% (3.24) groups showed significant elevation in their estimated level with the absence of difference between the two concentrations.

### Evaluation of VEGF1 and caspase 3 gene expression

**Fig. 4:**
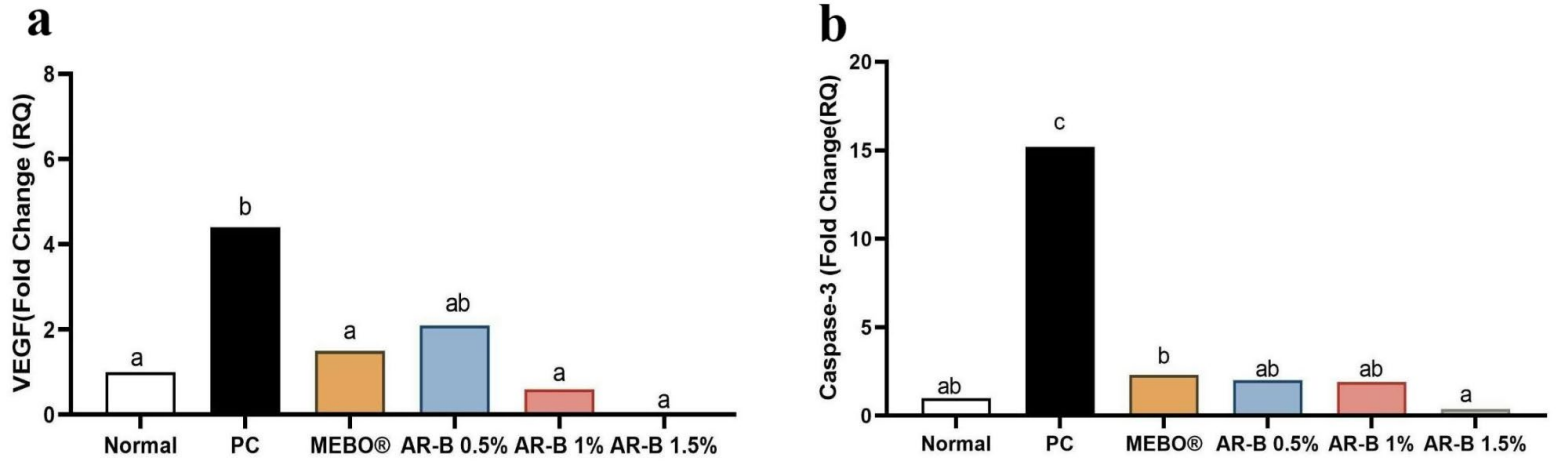
Quantitative RT–PCR of VEGF 1 and Caspase 3 gene expression in skin tissue in different experimental groups. mRNA relative expression of VEGF 1 (**a**) and Caspase 3 genes (**b**). a, b and c above the columns indicate significant difference at P˂0.05. The columns of the same letter have no significant difference in-between

As illustrated in Fig. 4. The lowest VEGF expression was recorded in the AR-B 1.5% group, followed by AR-B 1%. There was no statistically significant difference between these groups compared to the normal or MEBO® groups. On the other hand, the PC group showed a significant increase in VEGF expression compared to all groups except the AR-B 0.5% group.

The group that received the highest dose of AR-B showed the lowest apoptotic activity, with a significant decrease in caspase 3 level compared to the PC group. Low and medium doses-treated groups showed a higher expression in caspase 3 compared to AR-B 1.5%.

### Histopathology

**Fig. 5:**
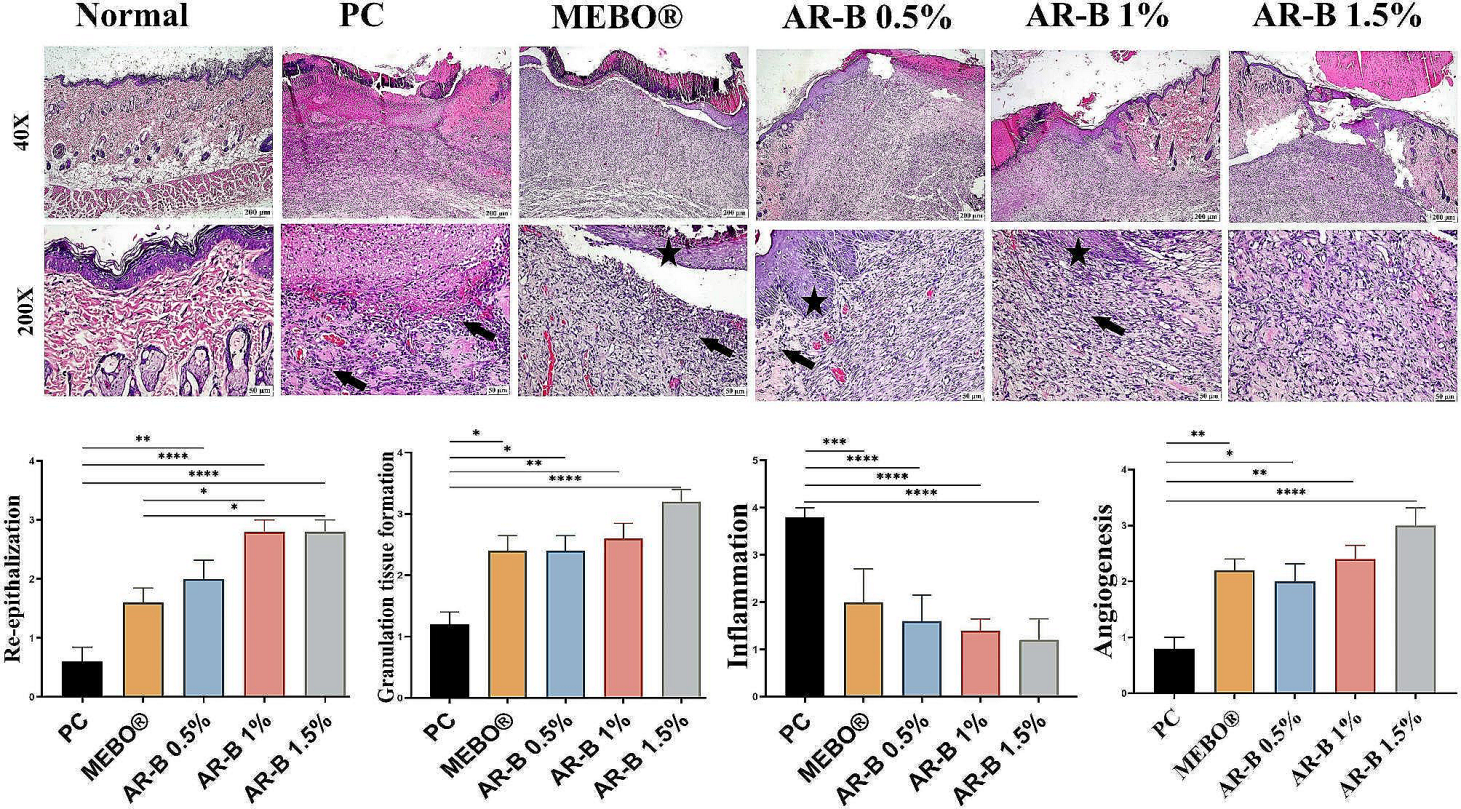
Photomicrographs of skin (H&E) showing the wound area in the different experimental groups after 7 days of induction. Inflammatory cells (arrows) and re-epithelization (stars). Charts represent the histological scores of groups on the 7th day. Data Expressed as means ± SE. Significant difference is considered at *P* < 0.05. (*) = summary of significance level and (ns) = non-significant

On the 7th day of wound induction (Fig. 5), Normal histological skin characteristics were observed during the examination of the normal group. Multiple epithelial layers were detected on the intact basement membrane at the epidermal layer. All were covered with normal thickness of keratin. The normal amount of fibrous tissue was found at the dermal layer along with skin adnexal tissue, including hair follicles, sweat, and sebaceous glands.

PC group displayed a significant wound gap. The whole epithelium was denuded and covered with a sero-cellular crust composed of necrotic cells, exudate, and inflammatory cells, mainly neutrophils. This gap was filled with a minimal amount of haphazardly arranged granulation tissue, which had many active fibroblasts, and a significant infiltration of inflammatory cells was detected. Few newly formed blood capillaries were observed. Additionally, bleeding was frequently seen.

The MEBO® treated group demonstrated partial re-epithelization with hyperplastic epithelium at the margins of the wound’s gap; the sero-cellular crust covered the remaining gap. The granulation tissue that filled the wound’s gap tended to be more regular, with little collagen at the wound’s base, and fewer neutrophils and lymphocytes were infiltrating the wound area. New blood capillaries were formed, oriented perpendicularly on the granulation tissue, especially toward the base. There were also many blood capillaries beneath the wound’s crust.

The group treated with AR-B showed 0.5% improvement in the histopathological alterations. The wound’s surface was covered with a thinner crust with less neutrophil and necrotic cellular debris content. Its edges exhibited partial re-epithelization at a better condition. The wound gap was filled with moderately inflamed granulation tissue, which tends to have a better orientation. Collagen bundles were more prominent in this group.

Wound healing was significantly improved in groups treated with higher concentrations of AR-B. The crust in the AR-B 1% treated group was even thinner, and neutrophils were smaller than those in the AR-B 0.5% group. Re-epithelization was still at the wound’s edges. Granulation tissue was better, and inflammation was moderate. Collagen was more abundant and admixed with highly active fibroblast. Additionally, there was a noticeable increase in newly formed blood capillaries.

Re-epithelization at the AR-B 1.5% group was comparable to AR-B 1%. The formed granulation tissue was abundant, progressing to form organized tissue. The collagen fibers were more prominent, and the wound gap appeared to be the most contracted among all groups. The degree of inflammation was minimal, while the newly formed blood capillaries were much more noticeable.

Histological tissue scoring (Fig. 5) revealed a significant improvement in re-epithelization in groups treated with higher doses of AR-B (1-1.5%). This improvement was even better than that observed with the MEBO® group. Granulation tissue and angiogenesis were also significantly increased in the AR-B 1.5% group.

**Fig. 6:**
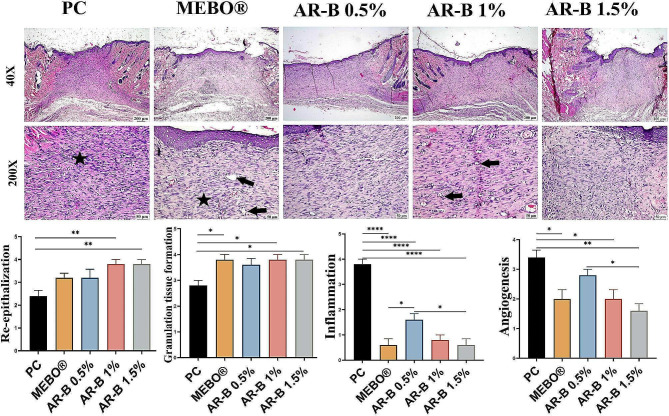
Photomicrographs of skin (H&E) showing the wound area in the different experimental groups after 14 days of induction. Newly formed capillaries (arrows) and inflammatory cells (star). Charts represent the histological scores of groups on the 14^th^ day. Data Expressed as means ± SE. Significant difference is considered at *P* < 0.05. (*) = summary of significance level and (ns) = non-significant

On the 14^th^ day (Fig. 6), a thin layer of newly formed epithelium was covered in the wound surface in the PC group. The wound gap was filled with well-vascularized granulation tissue with low collagen deposition. This group had the highest inflammatory cell infiltration.

MEBO® group experienced a significant improvement in re-epithelization with a moderate thickness of the formed epidermis. The tissue gap was filled with organized tissue with abundant collagen. The inflammation subsided with a few newly formed blood capillaries.

The wound treated with AR-B 0.5% showed a moderately thin regenerated epithelium. The gap was also filled with organized tissue. Compared to the MEBO® and other AR-B treated groups, the inflammation and angiogenesis were higher in this group.

The thickness of the regenerated epithelium at the AR-B, 1% group, was moderate. Organized connective tissue with thick collagen bundles filled the wound, resulting in a marked reduction in the wound size. The inflammation was minimal, with a significant decrease in the number of blood capillaries.

The best histological characteristics of wound healing were observed in the AR-B 1.5% group. A regenerated epithelium with high thickness was covering the wound surface. The wound gap was significantly contracted and filled with fully mature organized tissue merged with thickened collagen bundles. The inflammation and blood capillaries were minimal.

Based on the histologic scoring on the 14^th^ day (Fig. 6), it was observed that AR-B 1.5% showed the best healing characteristics among all treated groups. Similarly, AR-B 1% showed perfect re-epithelization and granulation tissue formation. However, the least inflammation and angiogenesis were reported in the AR-B 1.5% groups.

**Fig. 7:**
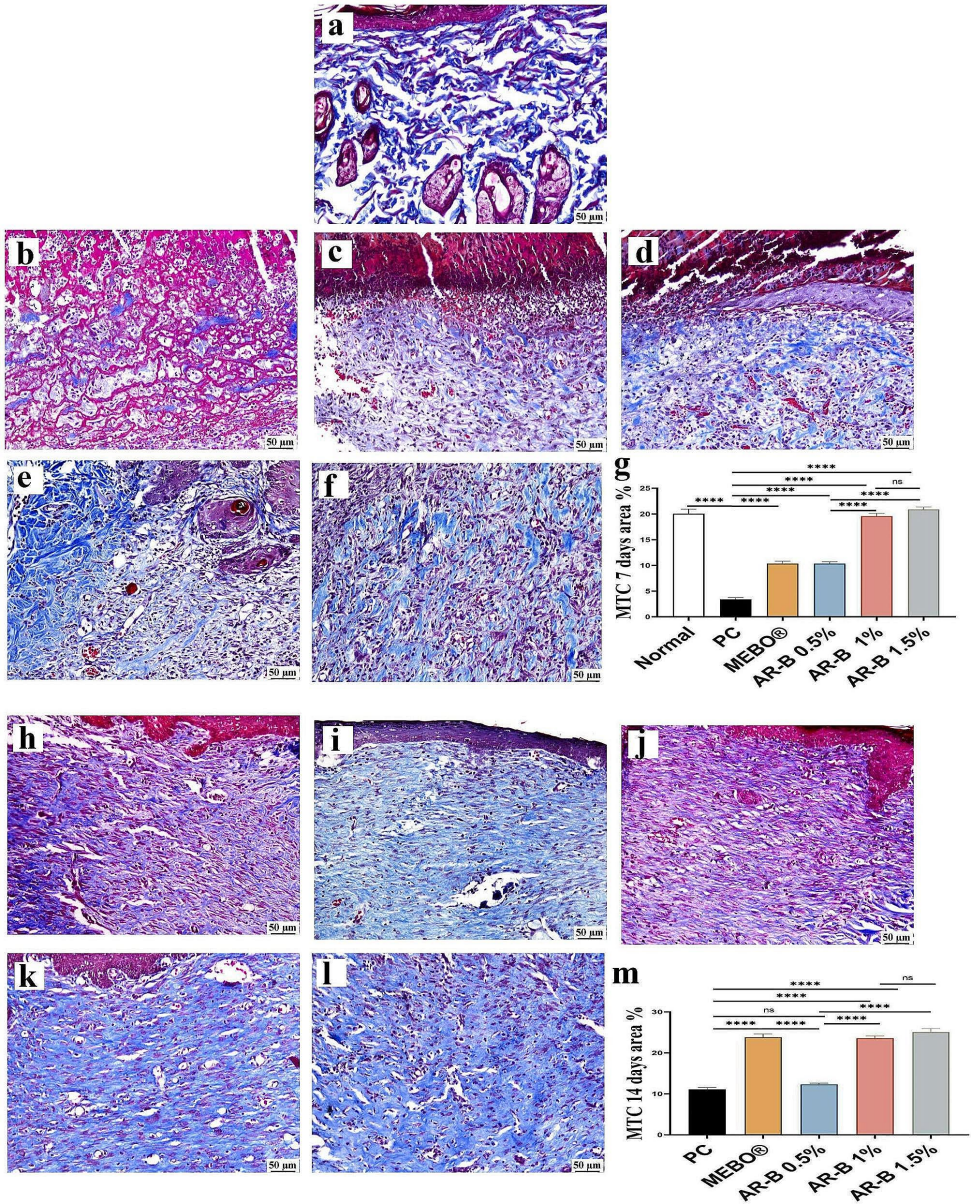
Photomicrographs of skin (MTC) on the 7^th^ day of wound induction (**a**) Normal group (**b**) PC, (**c**) MEBO®, (**d**) AR-B 0.5%, (**e**) ARB-1%, (**f**) AR-B 1.5% and on the 14^th^ day of induction (**h**) PC, (**i**) MEBO®, (**j**) AR-B 0.5%, (**k**) ARB-1%, (**l**) AR-B 1.5%. (**g** and **h**). Collagen fibers are blue stained. Charts present area % of MTC stain. Data are presented as means ± SE. Significant difference is considered at *P* < 0.05. (*) = summary of significance level and (ns) = non-significant

MTC stained sections were used to evaluate collagen deposition in the wounds’ gap of different groups on the 7^th^ and 14^th^ day (Fig. 7). On the 7^th^ day, there was a significant increase in collagen deposition in the groups treated with AR-B 1% and 1.5% compared to PC, MEBO®, and AR-B 0.5%. However, on the 14th day, collagen bundles were more extensive, profuse, and efficient in the groups treated with AR-B 1% and 1.5%. These groups were very similar to the MEBO® treated group.

### Immunohistochemistry

#### TGF-β expression

**Fig. 8:**
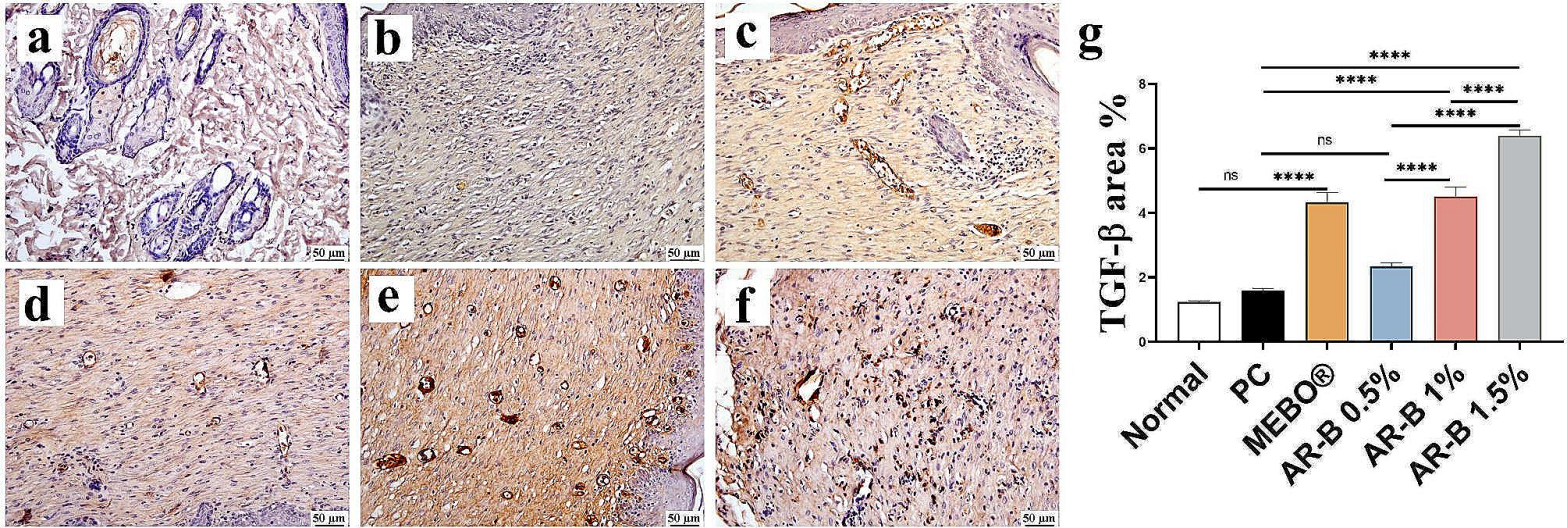
Photomicrographs of skin (Immune staining) showing TGF-β expression (**a**) Normal group, (**b**) PC (**c**) MEBO (**d**) AR-B 0.5% (**e**) AR-B 1% (**f**) AR-B 1.5% and (**g**) Chart represents TGF-β quantification as area percentage. Data are presented as mean ± SE. Significant difference is considered at *P* < 0.05. (*) = summary of significance level and (ns) = non-significant

All treated groups with AR-B exhibited a significant upregulation of TGF-β in a dose-dependent manner compared to the PC group. The highest expression of TGF-β was recorded in the AR-B 1.5% group, whereas the AR-B 1% group showed no significant difference compared to the MEBO® treated group (Fig. 8).

#### α-SMA expression

**Fig. 9:**
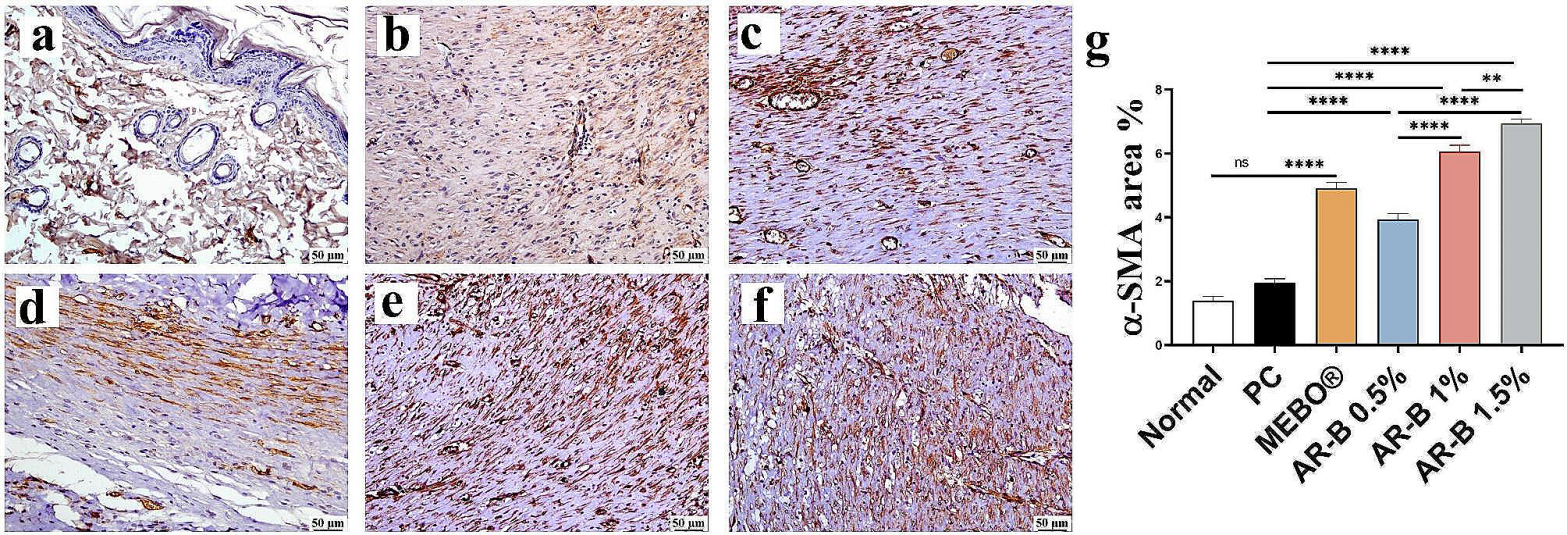
Photomicrographs of skin (Immune staining) showing α-SMA expression (**a**) Normal group, (**b**) PC (**c**) MEBO (**d**) AR-B 0.5% (**e**) AR-B 1% (**f**) AR-B 1.5% and (**g**) Chart represents α-SMA quantification as area percentage. Data are presented as mean ± SE. Significant difference is considered at *P* < 0.05. (*) = summary of significance level and (ns) = non-significant

Compared to the PC group, all treated groups exhibited a significant increase in α-SMA in a dose-dependent manner. The highest expression was observed in the AR-B 1.5% group (Fig. 9).

#### TNF-α expression

**Fig. 10:**
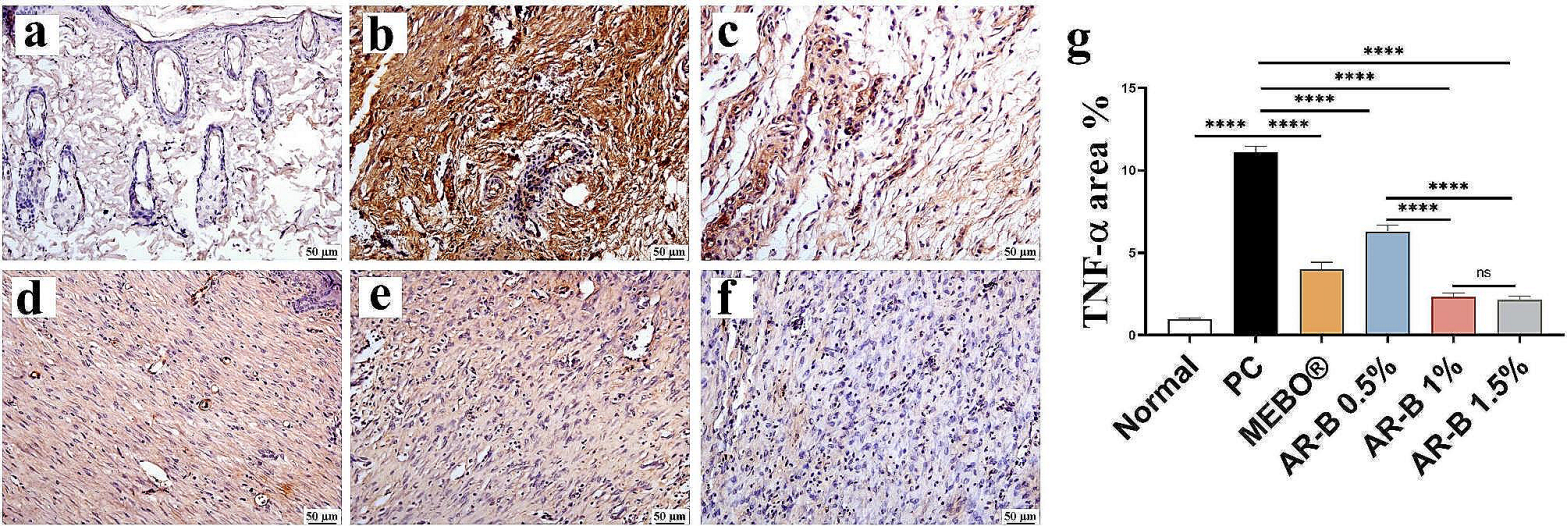
Photomicrographs of skin (Immune staining) showing TNF-α expression (**a**) Normal group, (**b**) PC (**c**) MEBO (**d**) AR-B 0.5% (**e**) AR-B 1% (**f**) AR-B 1.5% and (**g**) Chart represents TNF-α quantification as area percentage. Data are presented as mean ± SE. Significant difference is considered at *P* < 0.05. (*) = summary of significance level and (ns) = non-significant

Medium and high doses of AR-B (1% and 1.5%) significantly reduced TNF-α expression, with no significant difference in between. However, the PC untreated group significantly increased TNF-α expression (Fig. 10).

## Discussion

A wound is an injury that results in the body’s cells breaking down due to external assault, such as trauma, burns, accidents, and diseases [17]. The goalof wound healing is to return the cellular structure of damaged tissues to their original state of health through a coordinated process with numerous dynamic phases. Some researchers mentioned that the healing process could be accomplished in three phases: the inflammatory phase, proliferative phase is the second stage of healing, and re-epithelialization, fibroplasia, angiogenesis, and granulation tissue formation. The remodeling phase, the final stage of wound healing, involves the synthesis of modest amounts of scar tissue and the synthesis of collagen [18, 19].

Many plants, like *Caralluma* species, contain active compounds called pregnane glycosides. It has been found to have anti-inflammatory properties [20] and wound-healing activity for some steroid glycosides [21–23] Therefore, they could be promising compounds that promote wound healing. This study focuses on the effects of arabinoside B, a newly isolated pregnane glycoside from *Caralluma arabica*, on wound healing.

Pregnanes and pregnane glycosides are well-studied secondary metabolites, many of them exhibit anti-inflammatory, antibacterial, and wound healing properties [13]. For example, *Solenostemma argel* (Del.), a plant known for its rich content of pregnanes and pregnane glycosides, exhibits significant potential as a wound healing agent. These bioactive compounds contributed to the reduction of wound surface area, wound contraction, enhancement of re-epithelization, fibroblast proliferation and modulation of inflammation [24].

The isolated pregnane glycosides, namely russelioside A, russelioside B, russelioside C, and russelioside D, from *Caralluma quadrangula*, have demonstrated significant potentials in inhibiting the biofilm attachment and reducing bacterial loads making them promising candidates for the development of topical antimicrobial preparations to promote wound healing [25]. Additionally [26], demonstrated the anti-inflammatory action of russelioside A, a pregnane glycoside isolated from *Caralluma tuberculate*.

We used sodium carboxymethyl cellulose (NaCMC), which is a natural polymer; it exhibits promising attributes that consolidate its use as a topical application, aside from being a cost-effective material [17, 27].

Antioxidants are elements that aid in wound healing by donating their own electrons to other substances, such as ROS, converting them into more patent materials like H_2_O and O_2_. There are two types of antioxidants: non-enzymatic antioxidants, such as vitamin E, vitamin C, glutathione, and flavonoids, and enzymatic antioxidants, such as superoxide dismutase, catalase, and glutathione peroxidases [28].

Our results supported the role of AR-B as a potential antioxidant that protects the tissue against oxidative damage. That was evidenced by the decreased MDA levels in groups treated with AR-B, an intermediate product formed from lipid peroxidation [2]. These findings are consistent with the results reported by [15] who also noted the beneficial effects of AR-B.

Another short-lived free radical called nitric oxide (NO) is synthesized in wounds. Nitric oxide is related to the synthesis and contractile ability of collagen. As wounds age, there is a marked decrease in nitric oxide levels, as reported in [29]’s study. Our results pointed to a significant reduction in NO levels on the 14^th^ day of wound healing in AR-B treated groups, which could be attributed to AR-B being one of the pregnane glucosides [30].

SOD is an enzyme that protects against oxidative stress. During acute wound healing, SOD level decreases because it is consumed in the lipid peroxidation process. Initially, the SOD level decreases until the 7th day; after that, it starts to increase and reaches normal levels by the 14th day. Our study confirms this result, showing a significant increase in SOD levels in groups treated with AR-B, with the highest expression being recorded in the AR-B 1.5% group.

During the wound-healing process, reepithelization occurs due to the presence of keratinocytes. Measuring its efficiency is based on the degree of wound gap coverage and its thickening. Tissue fibers, collagen, and other extracellular matrices are established. These features are influenced by inflammatory cell formation, migration, and development of new blood capillaries between fibrous connective tissue fibers [31]. These are the four features that are the key to evaluating the healing process.According to [7], greater epithelial coverage and thickening indicate a higher degree of wound healing. In all groups treated with AR-B, reepithelization increased in a dose-dependent manner.

The main component of the extracellular matrix is collagen, and the more collagen deposition in the wound gap, the more advanced the wound repair is [32]. Collagen formation starts between 3 and 6 days, and its density increases over time [33]. We observed a noticeable increase in the expression of both TGF-β and α -SMA on the 14^th^ day of wound induction in both 1% and 1.5% AR-B treated groups. TGF-β is responsible for α -SMA activation, which activates collagen fibers proliferation and extracellular matrix deposition, causing wound contraction [34].

TNF-α plays a crucial role in the healing process by modulating various cytokines, and it is used to assess the inflammation degree during the wound repair process [7]. As mentioned by [35], faster wound healing requires a decrease in TNF-α levels; our findings showed that AR-B treatment significantly decreased TNF-α levels at an advanced stage of wound healing. This confirms the effect of AR-B in reducing inflammation during wound healing. The anti-inflammatory properties of AR-B could be attributed to the main criteria of pregnant glycoside, as previously described by [30, 36].

During wound healing, the formation of fibrovascular tissue is an important hallmark. By the 3rd day of wounding, the newly formed blood capillaries had started to appear. VEGF factor and angiogenesis processes drop, and the endothelial cells initiate apoptosis once the granulation tissue is produced. These steps are crucial to forming a hypocellular scar [37, 38]. Our findings align with the prior studies, as we demonstrated a significant reduction in the VEGF expression and the number of newly formed blood capillaries on the 14^th^ day of wounding in the groups treated with AR-B. During the later stages of wound healing, angiogenesis is suppressed due to the restoration of tissue hypoxia, decreased inflammation and growth factors. Pericytes secrete an inhibitory mediator, which inhibits vascular proliferation [38]. Our results confirm the efficacy of AR-B, as a new pregnane glycoside, in reducing inflammation VEGF and facilitating wound healing.

Apoptosis is a natural mechanism of cell death that both extrinsic and intrinsic factors could prompt. Caspase 3 factor is implicated in the stage of complete apoptosis of cells [39]. Our study found that the expression of caspase 3 was significantly decreased in groups treated with AR-B. Therefore, our research confirms that AR-B can potentially reduce tissue damage and promote healing.

## Conclusion

Our results highlighted the potential benefits of arabincoside B as a new pregnane glycoside isolated from *Caralluma arabica* in promoting skin wound healing as a topical application. Its mode of action could be attributed to its ability to improve re-epithelization, collagen deposition, anti-inflammation, and angiogenesis, which are crucial factors for proper wound healing. Also, this compound’s antioxidant and anti-apoptotic properties make it a good candidate for wound healing improvement. Further studies are needed to evaluate the formulated material for wound healing in different animal species.

## Methods

### Plant material

*Caralluma arabica* aerial parts were collected in July 2022 from the Aqan region, Al-Musaimir District, Lahej Governorate, Southern Yemen (13^°^ 22′ 871′′ **N**, 045° 83′ 344′′ **E**). Dr. Othman S. S. Al-Hawshabi, Associate Professor of Plant Taxonomy and Flora at the Department of Biology, Faculty of Science, Aden University, Yemen, collected and authenticated the plant material. The plant’s voucher specimen (No. 5659) was placed in the Department of Biology, Faculty of Science, University of Aden.

### Isolation

The aerial parts of *C. arabica* (300 g powder) were extracted with 95% ethanol following the method reported by [12, 13]. Part of the remaining water fraction (8 g) was subjected to chromatographic separation over flash silica gel column 60 (DCM-MeOH-H_2_O, 10:2:0.1) followed by separation of compound AR-B (450 mg) from fraction Fr-13 (1.03 g) by precipitation as white amorphous powder. The isolation was performed on flash silica gel 60 (Merck, particle size 230–400 mesh) and RP-C_18_ (silica gel, 40–63 μm; Merck). The spots were visualized after spraying with *p*-anisaldehyde/H_2_SO_4_ and heated at 110 °C.

### Gel formulation

#### Materials

Sodium carboxymethyl cellulose (NaCMC, average molecular weight 250,000 g/mol) was purchased from Sigma-Aldrich Co., St. Louis, USA. Propylene glycol, 95% ethanol, and sodium benzoate were procured from El-Nasr Pharmaceuticals (Cairo, Egypt).

#### Preparation of arabincoside B loaded NaCMC-based hydrogel

The Arabincoside B-loaded hydrogel was prepared under aseptic conditions by dispersing NaCMC (4% *w/v*) in propylene glycol in a porcelain dish. Propylene glycol acted as a dispersing agent to facilitate the formation of the 3D gel network. After that, the plant solid compound *(0.5 or 1%w/v*) was added to the propylene glycol dispersion. In a beaker, sodium benzoate (1% *w/v*), which acted as a preservative, was dissolved in water. Then, the propylene glycol dispersion was added gradually to the beaker containing water and placed on a hotplate stirrer adjusted at 60 °C and 100 rpm. Finally, the gel was transferred into sterilized vials and stored in the refrigerator till further use.

### Animals

Thirty-six male Wistar rats obtained from VACSERA (The Egyptian Company for producing vaccines, sera, and drugs) weighed between 200 and 230 g were kept in rats’ cages at optimum temperature conditions and lighting of 12-h light and dark cycle. They had *ad libitum* access to food and water.

After seven days of acclimatization, animals were divided randomly (using online randomization tool; http://www.randomizer.org) into 6 groups (6 animals/ group).

#### Group 1

(**Normal group**) as there was no induced wound.

#### Group 2

positive control group (**PC**) in which the wound was inducted and received no treatment.

#### Group 3

MEBO®-treated group in which MEBO® ointment (Herbal formulation containing β-sitosterol, baicalin, and berberine) was applied as a reference commercial drug.

#### Group 4

**AR-B -0.05%** group treated with AR-B -0.5% concentration.

#### Group 5

**AR-B − 1%** group treated with AR-B -1% concentration.

#### Group 6

**AR-B** -**1.5%** group treated with AR-B -1.5% concentration.

### Excisional wound induction and gross pathology

The skin wound was inducted under inhalation anesthesia with isoflurane according to the University of Pennsylvania’s IACUC guidelines of mouse anesthesia and analgesia recommendations [40]. The hair was removed, the hairless skin was then locally sterilized with 70% alcohol then punctured by 1 cm diameter biopsy punch to initiate 2 wounds on the dorsal aspect of each rat without breaking through any muscles. This study was performed according to the ethical guidelines and approved by the Institutional Animal Care and Use Committee in the Faculty of Veterinary Medicine, Cairo University, under the code (Vet CU09092023776).

Treatments were applied daily on the wounds for 14 days, and gross photos were taken at 0, 3, 7, 10, and 14 days to monitor wound closure and to measure the wound area. Image J 1.52 software was used to measure wound areas in 5 random wounds from each group on the time mentioned above intervals.

### Sample collection

Rats were euthanized according to the IACUC euthanasia guideline of the University of Iowa [41] using an overdose of isoflurane. The healed wound tissues were collected with scalpels and scissors. The collected samples were preserved in 10% neutral buffered formalin for histopathological examination (at two times intervals at 7 and 14 days), while the rest of the samples were collected at the end of the study (on the 14^th^ day) and assigned into two sets. The first set was homogenized in cold KCl buffer using a Teflon homogenizer, and the homogenates were centrifuged at 14,000×g for 20 min at 4 °C. The obtained supernatant was used to assess oxidant and antioxidant tissue biomarkers. The other set of samples was frozen immediately in liquid nitrogen and stored at -80 °C until the processing for RT-qPCR analysis.

### Oxidant and antioxidants analysis

The frozen wound tissue samples were washed with EDTA. After that, they were homogenized in a 1.15% KCl buffer and centrifuged at 4000 rpm for 10 min. The resulting supernatant was collected, and coulometric levels of malondialdehyde (MDA), superoxide dismutase (SOD), and nitric oxide (NO) were measured using a kit purchased from Biodiagnostic Company in Cairo, Egypt, according to the manufacturer’s instructions.

### Evaluation of VEGF1 and Caspase-3 gene expression by quantitative real-time PCR (qRT-PCR)

Total RNA was extracted from skin tissue with GF-1 Total RNA Extraction Kit according to the manufacturer’s instructions. The concentration and purity of the total RNA samples were obtained using a UNICO UV-2100 spectrophotometer. The isolated RNA was used for cDNA synthesis using M-MLV reverse transcriptase (Fermentas, EU). Real-time PCR (qPCR) was carried out using the reaction mixture of cDNAs, iQ SYBR Green Premix (Bio-Rad 170–880, USA), and 0.5mM of each primer (VEGF1, caspase 3 and B-actin as an internal control). PCR amplification and analysis were achieved using Bio-Rad iCycler thermal cycler and the MyiQ real-time PCR detection system. The primers and their sequence published in GenBank are shown in Table 1. Each assay includes triplicate samples of each tested cDNA and no-template negative control, and the ΔCT value is calculated according to [42] through the subtraction of the B-actin CT from each gene’s CT; in which CT is the cycle number where detectable signals are obtained.

**Table 1:** Primer Sequences of VEGF1, Caspase-3 and B-actin Genes

Gene	Primer	Sequence	Accession number	Amplicon (bp)	Ref
VEGF1	Forward	GCAATGATGAAGCCCTGGAG	*NM_001287111.1*	246	[43]
	Reverse	GCTTGTCACATACGCTCCAG			
Caspase 3	Forward	CATGCACATCCTCACTCGTG	*NM_012922.2*	158	[44]
	Reverse	CCCACTCCCAGTCATTCCTT			
B-Actin (reference gene)	Forward	AGGCTGTGTTGTCCCTGTATG	NM_031144.3	275	[45]
	Reverse	GGCCATCTCTTGCTCGAAGT			

#### Histopathological evaluation

Collected samples were preserved in 10% neutral buffered formalin, then dehydrated and embedded into paraffin blocks. They were sectioned into 5 μm sections, which were rehydrated for routine staining with hematoxylin and eosin (H&E) and Masson’s trichrome (MTC) using Masson Trichrome kit (MST-100T, Biognost, Croatia) as manufacturer instructions to demonstrate collagen fibers (blue stained) and quantified as (area %) in five random microscopic fields in each group using Cellsens dimensions (Olympus software) [46]. A Leica DM4 B light microscope (Germany) was used for tissue examination, and a Leica DMC 4500 digital camera (Germany) was used to capture images.

Wound healing scoring was based on the essential histological characteristics for wound healing mentioned by [33]. Each characteristic was scored from 0 to 4. First, reepithelization t (0 = no re-epithelialization, 1 = Poor re-epithelization, 2 = incomplete re-epithelization, 3 = moderate re-epithelization and 4 = complete re-epithelization). Secondly, granulation tissue formation and collagen association were scored as (0 = not fully formed granulation tissue, 1 = thin granulation tissue, 2 = moderate restoration, 3 = profuse granulation tissue, and well-formed collagen, and 4 = extensively organized tissue). Then, the degree of inflammation was scored by counting inflammatory cells per histological field (0 = there are 1–4 inflammatory cells per field, 1 = there are 4–7 inflammatory cells per field, 2 = there are 7–10 inflammatory cells per field, 3 = there are 10–13 inflammatory cells per field, and 4 = there are 13–15 inflammatory cells per field. Finally, we evaluated the level of angiogenesis per field as well as the presence of congestion, hemorrhage, and edema (0 = no angiogenesis with the existence of congestion, hemorrhage, edema, 1 = 1 − 2 vessels, edema, hemorrhage and congestion, 2 = 3–4 vessels, moderate edema and congestion, 3 = 5–6 vessels, slight edema and congestion, 4 = 7 vessels or more which are arranged vertically toward the epithelial surface). The mean score of each evaluated criteria was obtained from ten random microscopic fields representing each group.

### Immunohistochemistry

As described by [7]. Tissue blocks were sliced into 5 μm thick sections and placed on positively charged slides. After deparaffinization and rehydration, the slides were subjected to heat in a microwave to induce epitope retrieval. H_2_O_2_ was applied for 10 min to block endogenous peroxidase. The tissue sections were then washed with PBS and incubated for an hour with monoclonal mouse anti-antibodies, including anti-tumor necrosis factor α (TNF-α) (52B83; SC-52,746) (at a dilution of 1:200, Santa Cruz Biotechnology, Inc.), anti-alpha smooth muscle actin (α-SMA) (Ready-to-use, Scyteck, USA), and anti-transforming growth factor β (TGF-β) (3C11, SC-130,348) (at a dilution of 1:200, Santa Cruz Biotechnology, Inc.). After being washed again, a horse radish peroxidase-labeled secondary detection kit (BSB-0015, BioSB, USA) was used as manufacturer instructions to visualize the reaction. Finally, slides were stained with hematoxylin as a counterstain. To obtain negative control slides, the primary antibody was removed. Positive staining was quantified and expressed as the mean percentage of the area from five random high-power fields in each group using the CellSens dimensions software (Olympus).

### Statistical analysis

The statistical data in this study was presented using the standard error of the mean (SEM) and Graph Prism program. The ordinary one-way ANOVA test was used to test the significant differences between the tested groups. Differences were considered significant when *P* < 0.05.

### Electronic supplementary material

Below is the link to the electronic supplementary material.

Supplementary Material 1

## Data Availability

All data used during the current study is available in the article.

## Abbreviations

AR-B: Arabincoside B
C. *arabica*: *Caralluma arabica*
PC: Positive control
SOD: Superoxide dismutase
NO: Nitric oxide
MDA: Malondialdehyde
VEGF: Vascular endothelial growth factor
α-SMA: Alpha smooth muscle actin
TGF-β: Transforming growth factor β
TNF-α: Tumor necrosis factor α
PDGF: Platelets derived growth factor
NaCMC: Sodium carboxymethyl cellulose
g: Gram
mg: Milligram
UK: United Kingdom
MHz: Megahertz
NY: New York
USA: United States of America
ESI: Electrospray ionization
CC: Column chromatography
nm: Nanometer
VACSERA: The Egyptian Company for the production of vaccines, sera, and drugs
IACUC: Institutional Animal Care & Use Committee
RT-qPCR: Reverse transcription-quantitative real-time Polymerase chain reaction
KCl: Potassium chloride
RNA: Ribonucleic acid
H&E: Hematoxylin and eosin
MTC: Masson’s trichrome
µm: Micrometer
Vet-IACUC: Faculty of Veterinary Medicine ethical committee
EU: European Union
UV: Ultraviolet rays
