# Supplementary material for: Enhanced wound healing potential of arabincoside B isolated from Caralluma Arabica in rat model; a possible dressing in veterinary practice
Source: BMC Vet Res. 2024 Jun 29;20:282. doi: 10.1186/s12917-024-04128-2 (PMC11218188; doi:10.1186/s12917-024-04128-2)
Supplement: Supplementary file 1 — Supplementary Material 1 [file 12917_2024_4128_MOESM1_ESM.docx]

**Table (1.S) ^1^H- and ^13^C-NMR spectral data of arabincoside B (DMSO-d_6_)**

|  | **^1^H-NMR** | **^13^C-NMR** |  | **^1^H-NMR** | **^13^C-NMR** |
| --- | --- | --- | --- | --- | --- |
| **No.** |  |  | **No.** | **Dig** | |
| 1 | 1.01 (1H, *m*), 1.80 (1H, *m*) | 37.18 | 1` | 4.20 (1H, *d*, *J*= 7.60) | 101.75 |
| 2 | 1.48 (2H, *m*) | 29.76 | 2` | 3.58 (1H, *m*) | 69.11 |
| 3 | 3.40 (1H, *m*) | 77.58 | 3` | 3.07 (1H, *m*) | 84.49 |
| 4 | 1.52 (1H, *m*), 2.35 (1H, *dd*, *J*= 2.84, 10.48) | 38.71 | 4` | 4.05 (1H, *b.s*) | 74.00 |
| 5 | - | 139.66 | 5` | 3.52 (1H, *m*) | 69.75 |
| 6 | 5.35 (1H,  *br d, J*= 5.24) | 122.19 | 6` | 1.13 (3H, *d*, *J*= 6.2) | 17.62 |
| 7 | 1.74 (1H, *m*), 2.16 (1H, *m*) | 27.40 | OCH_3_ | 3.38 (3H, *s*) | 58.22 |
| 8 | 1.58 (1H, *m*) | 37.14 | **Glc** | | |
| 9 | 1.10 (1H, *m*) | 45.89 | 1`` | 4.28 (1H, *d*, *J*= 7.72) | 103.50 |
| 10 | - | 36.98 | 2`` | 2.95 (1H, *m*) | 74.63 |
| 11 | 1.32 (1H, *m*), 1.43 (1H, *m*) | 20.82 | 3`` | 3.05 (1H, *m*) | 77.19 |
| 12 | 1.34 (1H, *m*), 2.12 (1H, *m*) | 38.85 | 4`` | 3.07 (1H, *m*) | 70.92 |
| 13 | - | 49.07 | 5`` | 3.31 (1H, *m*) | 76.82 |
| 14 | - | 84.62 | 6`` | 3.97 (1H, *d*, *J*= 11.64), 3.58 (1H, *m*) | 69.47 |
| 15 | 1.93 (1H, *m*), 1.61 (1H, *m*) | 33.85 | **Glc** | | |
| 16 | 1.83 (1H, *m*), 1.93 (1H, *m*) | 23.79 | 1``` | 4.36 (1H, *d*, *J*= 7.68) | 103.92 |
| 17 | 2.72 (1H, *dd*, *J*= 4.8, 9.2) | 62.99 | 2``` | 2.98 (1H, *m*) | 73.96 |
| 18 | 0.88 (3H, *s*) | 15.70 | 3``` | 3.05 (1H, *m*) | 77.19 |
| 19 | 0.93 (3H, *s*) | 19.66 | 4``` | 3.02 (1H, *m*) | 70.52 |
| 20 | - | 215.61 | 5``` | 3.10 (1H, *m*) | 77.36 |
| 21 | 2.20 (3H, *s*) | 31.94 | 6``` | 3.67 (2H, *dd, J*= 5.6, 11.60) | 61.54 |
|  | **^1^H-NMR** | **^13^C-NMR** |  | **^1^H-NMR** | **^13^C-NMR** |
| **No.** |  |  | **No.** | **Dig** | |
| 1 | 1.01 (1H, *m*), 1.80 (1H, *m*) | 37.18 | 1` | 4.20 (1H, *d*, *J*= 7.60) | 101.75 |
| 2 | 1.48 (2H, *m*) | 29.76 | 2` | 3.58 (1H, *m*) | 69.11 |
| 3 | 3.40 (1H, *m*) | 77.58 | 3` | 3.07 (1H, *m*) | 84.49 |
| 4 | 1.52 (1H, *m*), 2.35 (1H, *dd*, *J*= 2.84, 10.48) | 38.71 | 4` | 4.05 (1H, *b.s*) | 74.00 |
| 5 | - | 139.66 | 5` | 3.52 (1H, *m*) | 69.75 |
| 6 | 5.35 (1H,  *br d, J*= 5.24) | 122.19 | 6` | 1.13 (3H, *d*, *J*= 6.2) | 17.62 |
| 7 | 1.74 (1H, *m*), 2.16 (1H, *m*) | 27.40 | OCH_3_ | 3.38 (3H, *s*) | 58.22 |
| 8 | 1.58 (1H, *m*) | 37.14 | **Glc** | | |
| 9 | 1.10 (1H, *m*) | 45.89 | 1`` | 4.28 (1H, *d*, *J*= 7.72) | 103.50 |
| 10 | - | 36.98 | 2`` | 2.95 (1H, *m*) | 74.63 |
| 11 | 1.32 (1H, *m*), 1.43 (1H, *m*) | 20.82 | 3`` | 3.05 (1H, *m*) | 77.19 |
| 12 | 1.34 (1H, *m*), 2.12 (1H, *m*) | 38.85 | 4`` | 3.07 (1H, *m*) | 70.92 |
| 13 | - | 49.07 | 5`` | 3.31 (1H, *m*) | 76.82 |
| 14 | - | 84.62 | 6`` | 3.97 (1H, *d*, *J*= 11.64), 3.58 (1H, *m*) | 69.47 |
| 15 | 1.93 (1H, *m*), 1.61 (1H, *m*) | 33.85 | **Glc** | | |
| 16 | 1.83 (1H, *m*), 1.93 (1H, *m*) | 23.79 | 1``` | 4.36 (1H, *d*, *J*= 7.68) | 103.92 |
| 17 | 2.72 (1H, *dd*, *J*= 4.8, 9.2) | 62.99 | 2``` | 2.98 (1H, *m*) | 73.96 |
| 18 | 0.88 (3H, *s*) | 15.70 | 3``` | 3.05 (1H, *m*) | 77.19 |
| 19 | 0.93 (3H, *s*) | 19.66 | 4``` | 3.02 (1H, *m*) | 70.52 |
| 20 | - | 215.61 | 5``` | 3.10 (1H, *m*) | 77.36 |
| 21 | 2.20 (3H, *s*) | 31.94 | 6``` | 3.67 (2H, *dd, J*= 5.6, 11.60) | 61.54 |

Arabincoside B

**Fig (1.S) ^1^H-NMR spectrum of arabincoside B (DMSO-d_6_)**

Arabincoside B

**Fig (2.S) ^13^C-NMR spectrum of arabincoside B (DMSO-d_6_)**
